# Synthesis of Natural Products Using Engineered Plants and Microorganisms

**DOI:** 10.3390/molecules29215054

**Published:** 2024-10-26

**Authors:** Yongjun Wei, Lingbo Qu, Xiaojun Ji

**Affiliations:** 1 School of Pharmaceutical Sciences, Zhengzhou University, Zhengzhou 450001, China; 2 Zhengzhou Research Base, State Key Laboratory of Cotton Bio-Breeding and Integrated Utilization, Laboratory of Synthetic Biology, Zhengzhou University, Zhengzhou 450001, China; 3 State Key Laboratory of Materials-Oriented Chemical Engineering, College of Biotechnology and Pharmaceutical Engineering, Nanjing Tech University, Nanjing 211816, China

Microorganisms and plants, particularly medicinal herbs, are abundant sources of diverse natural products, many of which are bioactive molecules with significant pharmaceutical or health benefits, and include artemisinin [1,2], ginsenosides [3], and *Ganoderic* triterpenes [4]. However, the contents of these bioactive compounds in their natural sources are often low [5]. The advancement of omics technologies and synthetic biology has opened new avenues for producing these valuable molecules through metabolically engineered plants and microorganisms [6]. Notable examples include the scaled-up production of artemisinin, rare ginsenosides, and various other natural products, which have potential applications in drugs, cosmetics, functional foods, animal feed, and other health-related products [7]. There is growing interest in utilizing cutting-edge synthetic biology technologies to further enhance the utilization of these natural products through the engineering of both plants and microorganisms [8].

This Special Issue is a collection of relevant research articles and reviews that highlight recent advancements in the production of bioactive natural products using engineered plants or microorganisms. Zhao, et al. summarized the artemisinin biosynthesis pathway, discussed advances in heterologous production using *Escherichia coli* and *Saccharomyces cerevisiae*, and explored metabolic engineering strategies in *Artemisia annua* and other plants to enhance artemisinin yields [9]. Zhang, et al. examined the pharmacological activities, biosynthesis pathways, extraction methods, and advances in the biotransformation and microbial synthesis of *Epimedium* flavonoids. Moreover, they discussed strategies to boost the industrial-scale production and utilization of *Epimedium* flavonoids [10]. Xu, et al. described the extraction, content determination, bioactivities, and product development of *Lycium ruthenicum* Murray’s bioactive substances, and discussed their potential medicinal and economic value [11]. Drożdżyński, et al. explored how plants and their endophytes adapt to harsh environments, focusing on the bioactive metabolites produced by them in synanthropic ruderal plants for potential use in agriculture and medicine [12]. Su, et al., using transcriptome and metabolome tools, revealed that the adaptation of *Citrus reticulata* ‘Chachi’ to a saline environment enhanced its high production of naringin and narirutin [13].

The function of bioactive natural products in plants and microorganisms are discussed in this Special Issue. Ma, et al. investigated the triterpenoids and ergostane steroids in *Ganoderma luteomarginatum,* which is distributed in Hainan province, China, and their evaluation of several natural products indicated that certain natural products displayed potential anti-tumor effects [14]. Alanazi, et al. found that *Syzygium aromaticum* (clove) oil had the ability to inhibit methicillin-resistant *Staphylococcus aureus* infection [15]. Sun, et al. identified and evaluated the anti-inflammatory constituents in Ardisiae Japonicae Herba (AJH) extracts, and found that ethyl acetate extract (EA) displayed significant anti-inflammatory activity and contained key pharmacodynamic components like bergenin, quercetin, and epigallocatechingallate, crucial for treating acute lung injury (ALI) [16]. Zhang, et al. found that ginsenoside Rb1, compound K, and 20(S)-protopanaxadiol had the ability to attenuate high-fat-diet-induced hyperlipidemia in rats by modulating the gut microbiota and bile acid metabolism [17].

Sun, et al. successfully increased the yield of 1,4-diaminobutane to 272 mg/L from engineered *E. coli* via the optimization of the synthesis of the cofactors PLP and NADPH [18]. Caballero Cerbon, et al. highlighted that eliminating L-cysteine desulfhydrases is key to improving L-cysteine production in engineered microbes, and outlined several strategies to tackle this issue [19]. Li, et al. demonstrated that biological nano selenium (SeNPs) and sodium selenite (Na_2_SeO_3_) treatments enhanced the organic selenium content in cowpea pods, and identified that a certain ABC transporter family played a crucial role in selenium absorption and transport [20]. Tu, et al. isolated *Klebsiella aerogenes* TL3 from termite guts, and demonstrated that it could degrade lignin anaerobically through a novel mechanism involving C-O and C-C bond cleavage, side chain oxidation, demethylation, and aromatic ring destruction [21].

Despite significant achievements in the synthesis of natural products using engineered plants and microorganisms, many bioactive natural products remain undeveloped [22]. Therefore, it is essential to leverage interdisciplinary tools, such as synthetic biology and engineering biology, to promote the development of related industries [23]. Through close collaboration between academia and industry, it is anticipated that a greater number of bioactive natural products could be discovered and utilized via engineered plants and microorganisms, thereby promoting technological innovation and market expansion in related industries.
